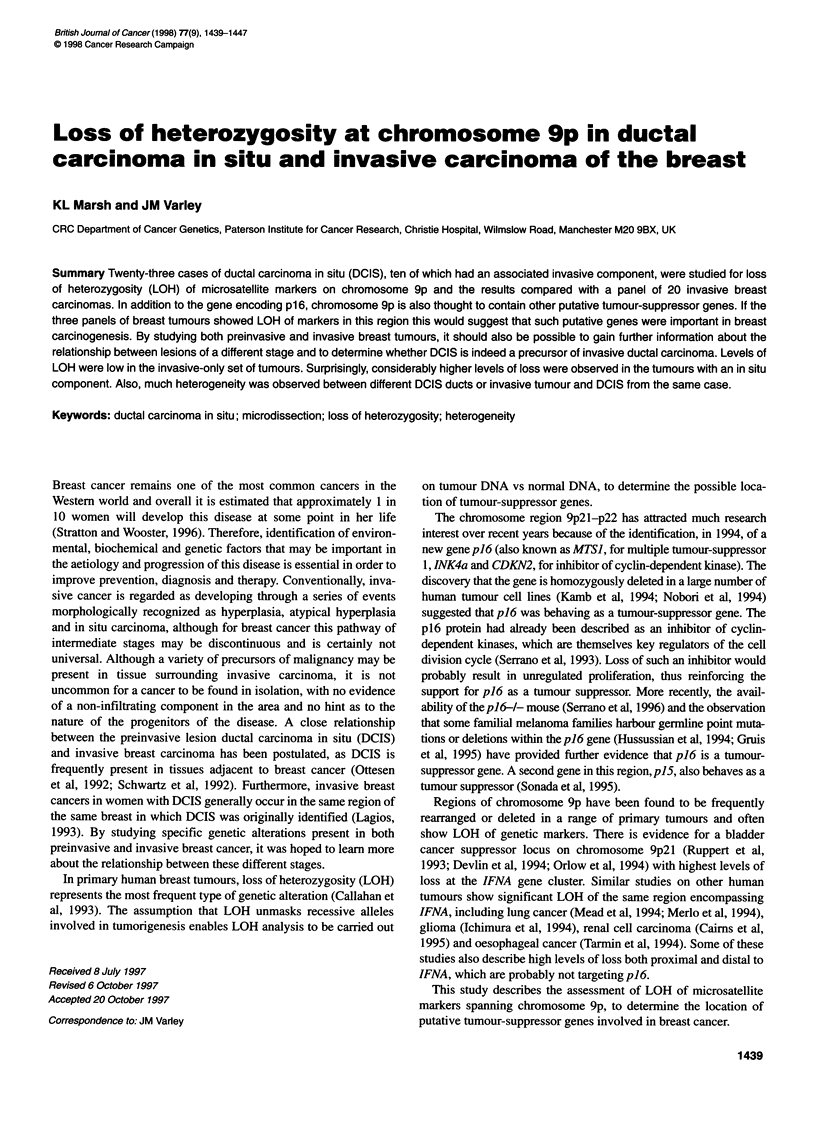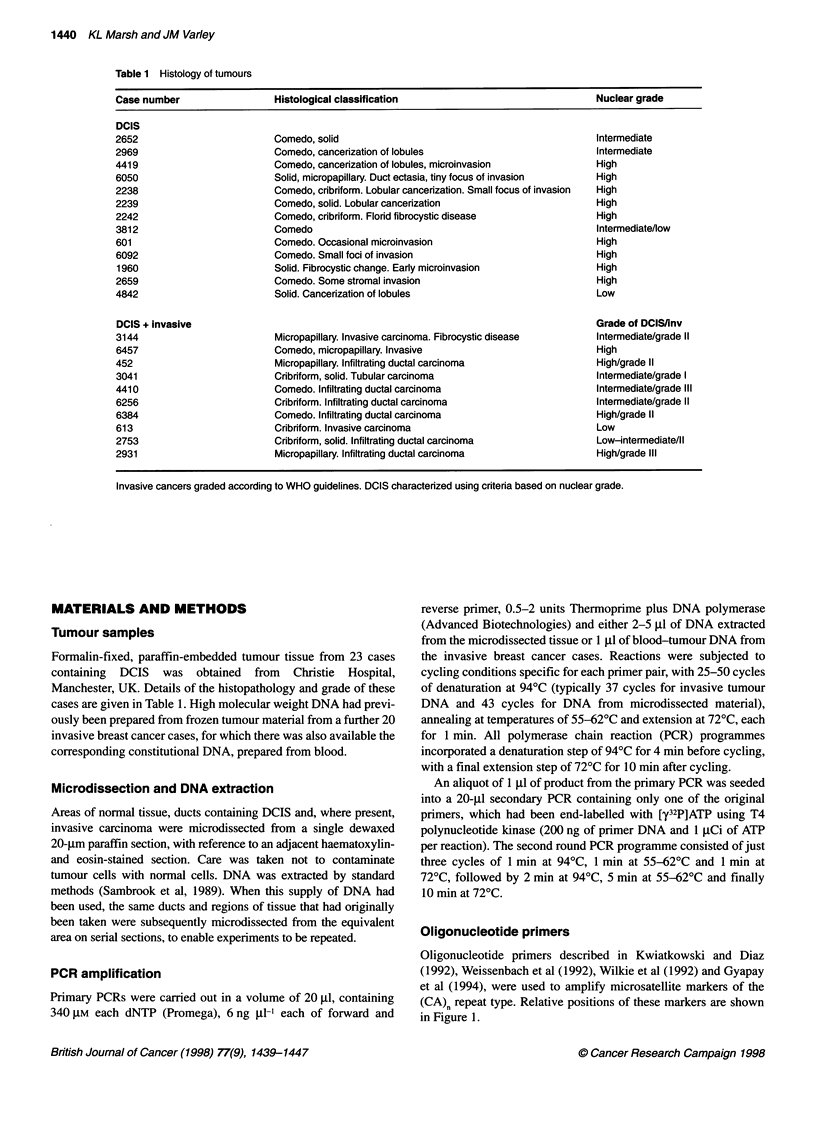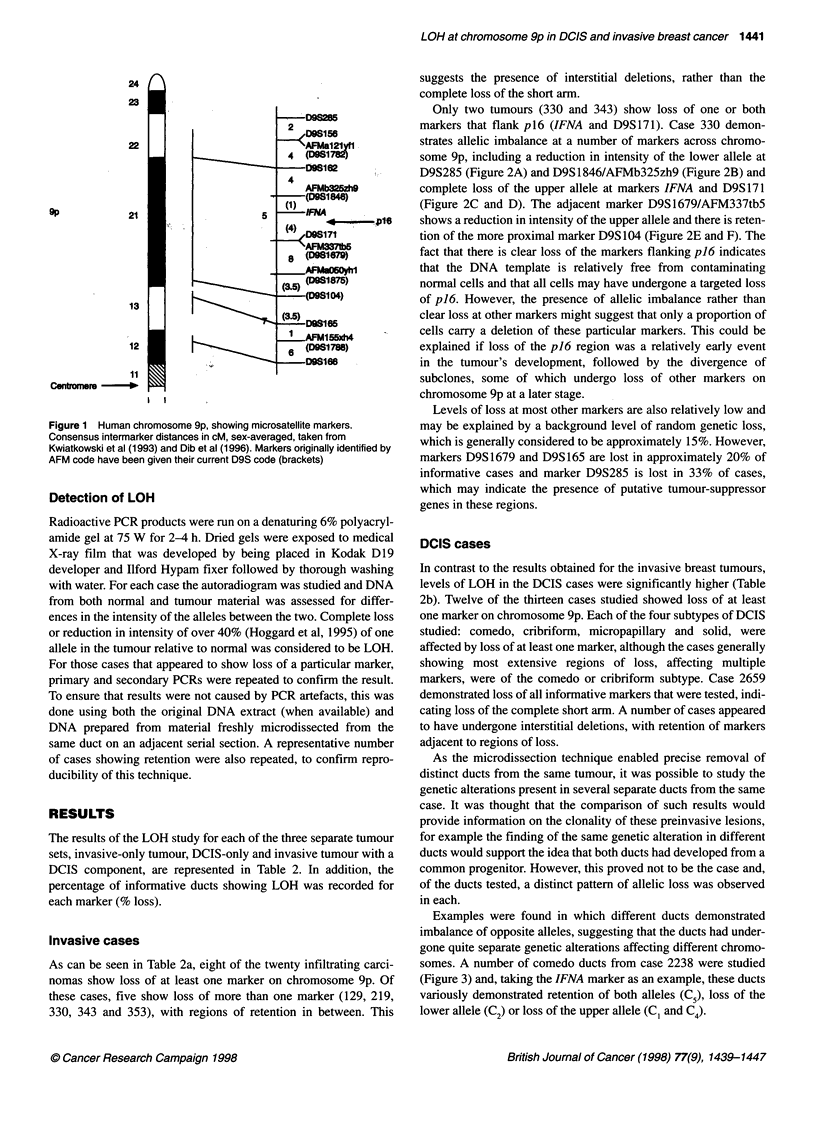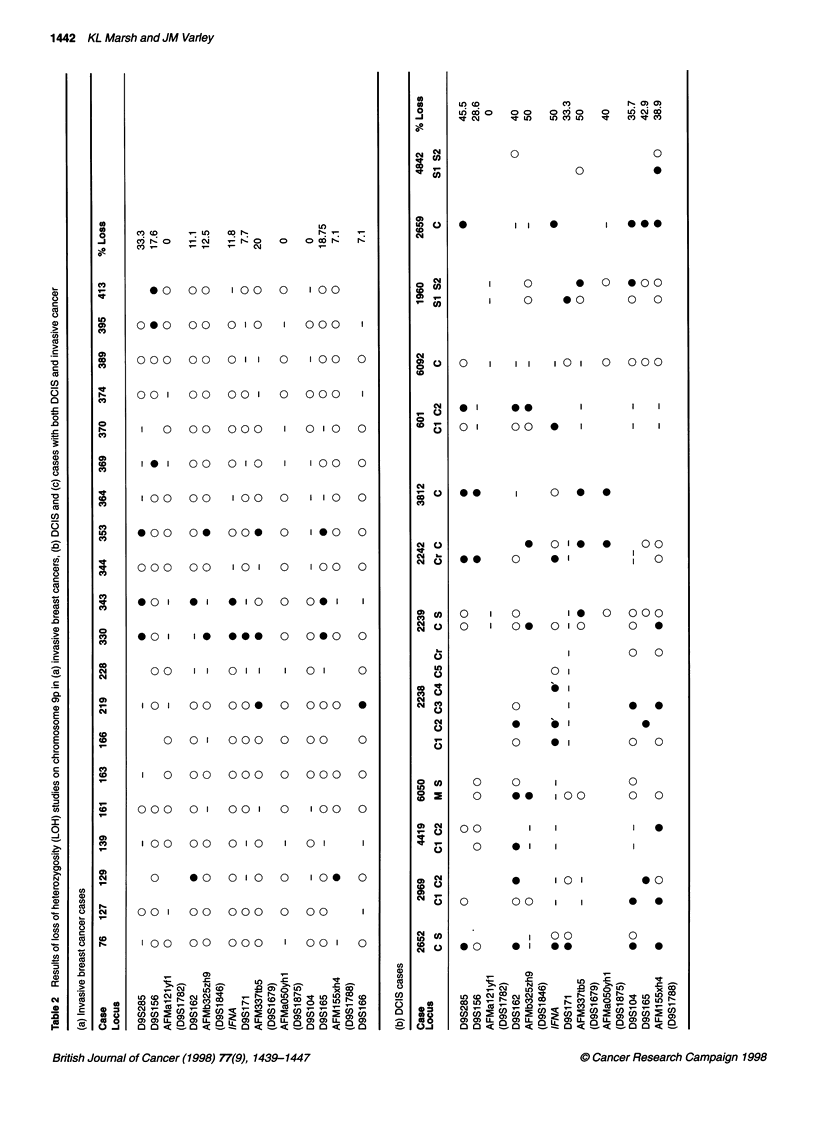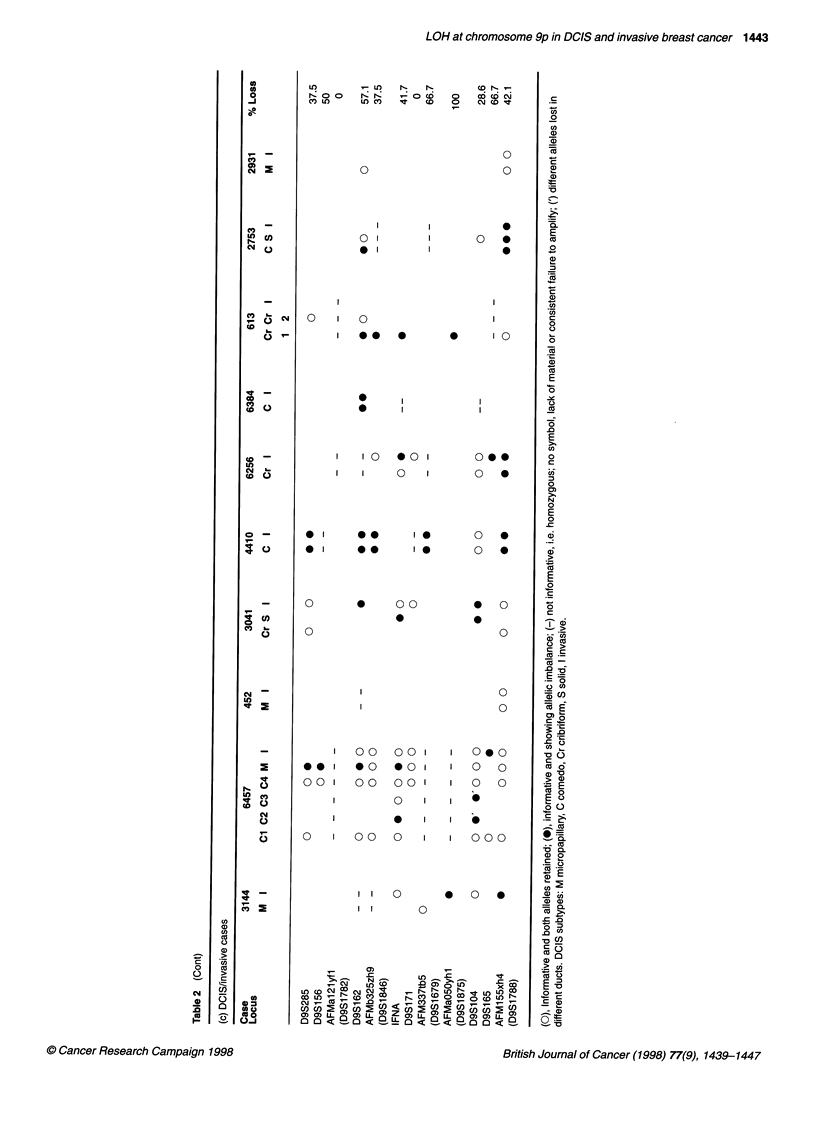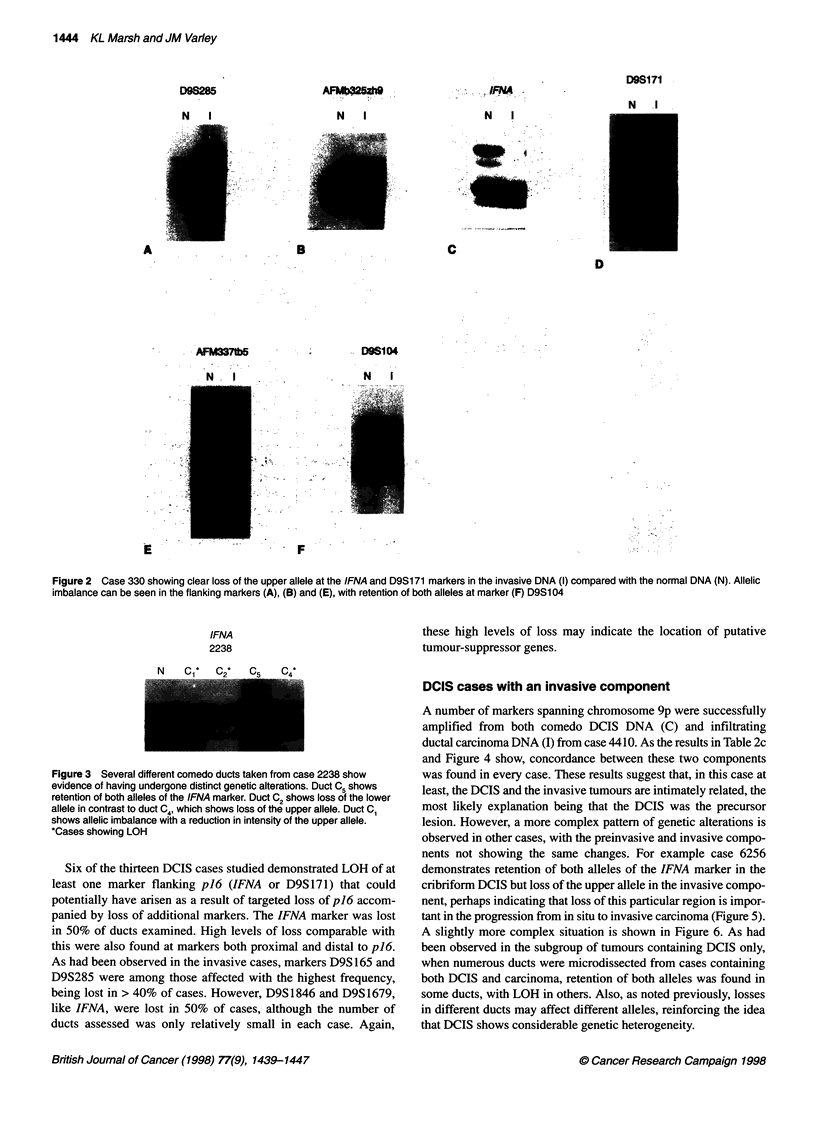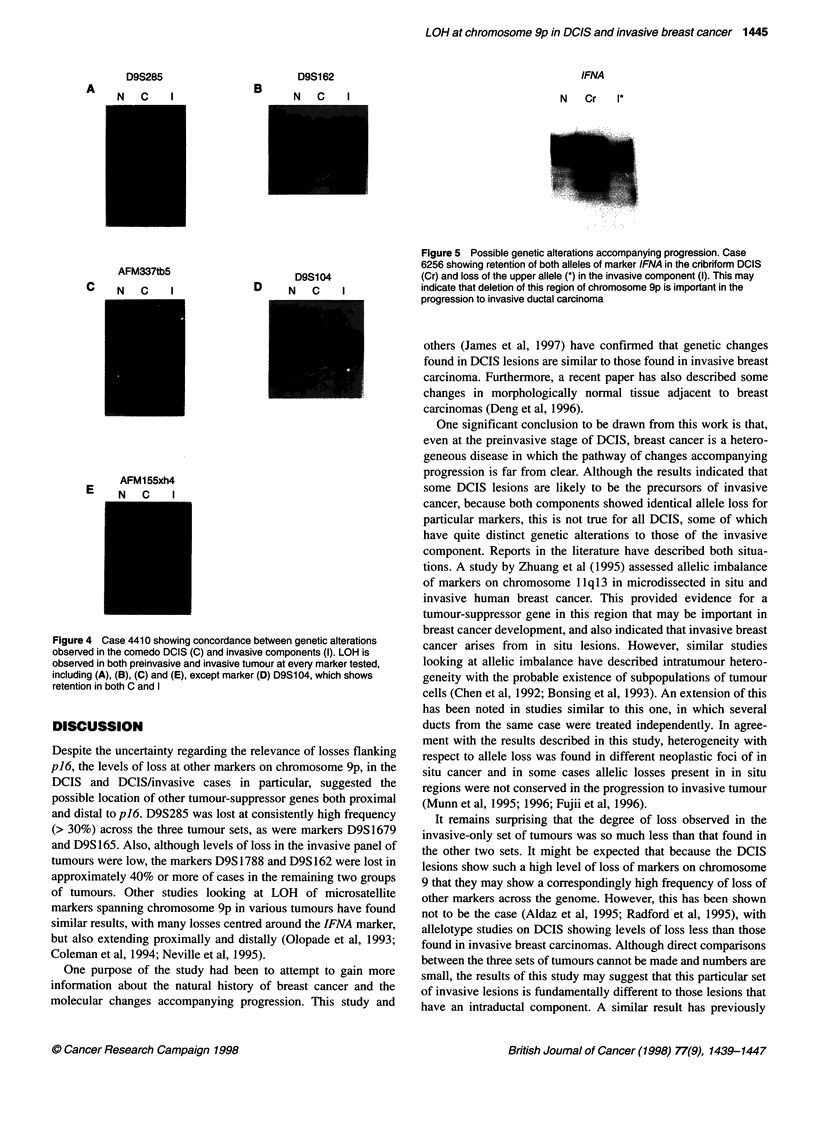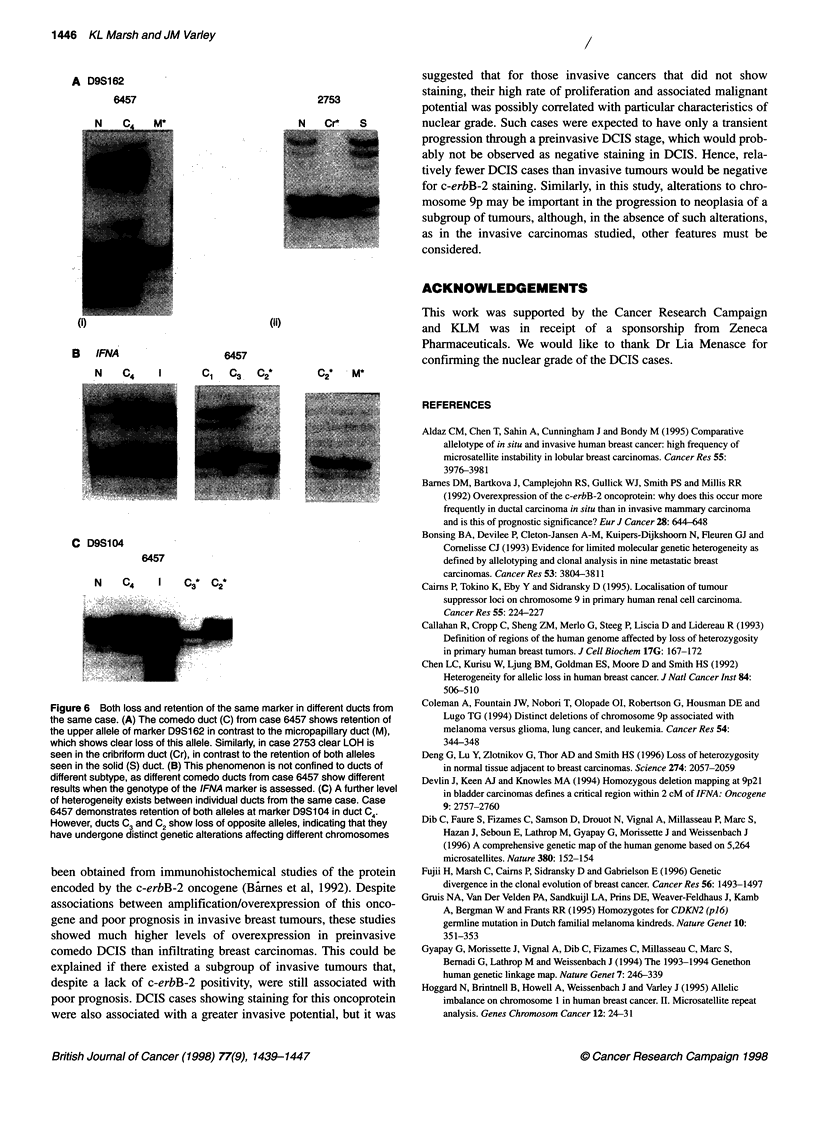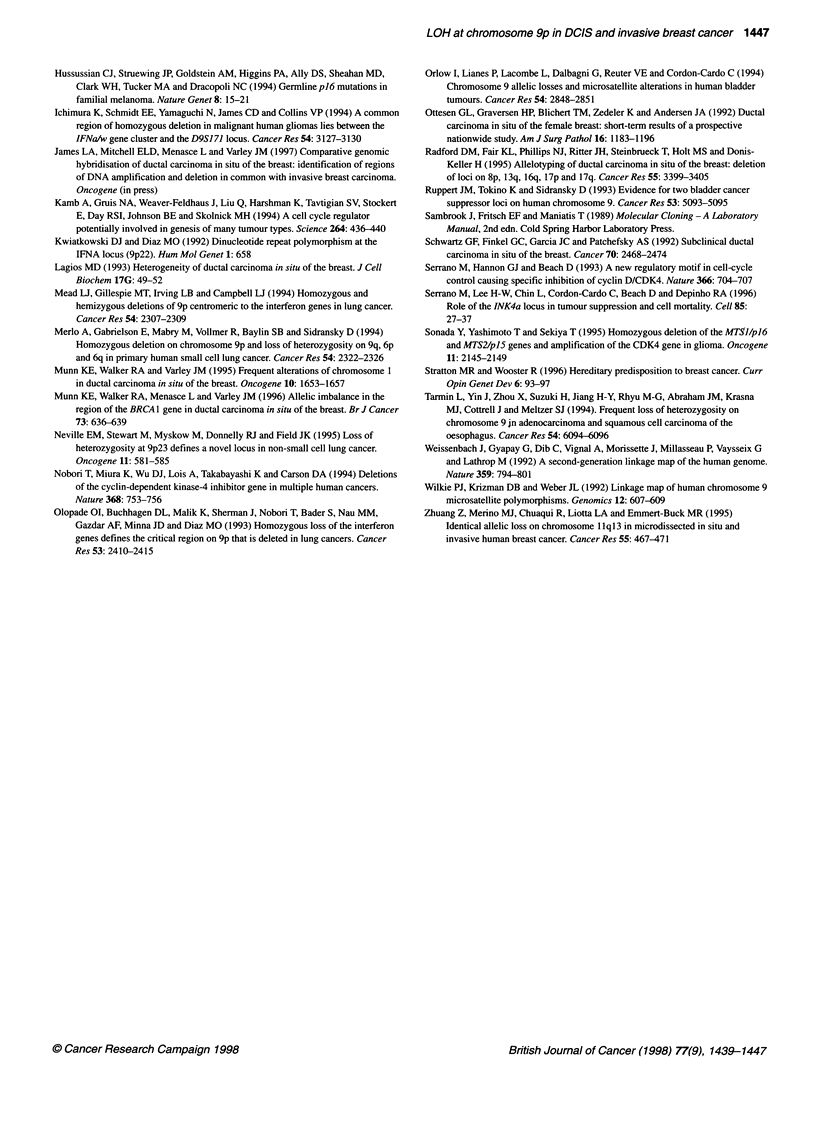# Loss of heterozygosity at chromosome 9p in ductal carcinoma in situ and invasive carcinoma of the breast.

**DOI:** 10.1038/bjc.1998.237

**Published:** 1998-05

**Authors:** K. L. Marsh, J. M. Varley

**Affiliations:** CRC Department of Cancer Genetics, Paterson Institute for Cancer Research, Christie Hospital, Manchester, UK.

## Abstract

**Images:**


British Journal of Cancer (1998) 77(9), 1439–1447
© 1998 Cancer Research Campaign

### Loss of heterozygosity at chromosome 9p in ductal carcinoma in situ and invasive carcinoma of the breast

**KL Marsh and JM Varley**

CRC Department of Cancer Genetics, Paterson Institute for Cancer Research, Christie Hospital, Wilmslow Road, Manchester M20 9BX, UK

**Summary** Twenty-three cases of ductal carcinoma in situ (DCIS), ten of which had an associated invasive component, were studied for loss of heterozygosity (LOH) of microsatellite markers on chromosome 9p and the results compared with a panel of 20 invasive breast carcinomas. In addition to the gene encoding p16, chromosome 9p is also thought to contain other putative tumour-suppressor genes. If the three panels of breast tumours showed LOH of markers in this region this would suggest that such putative genes were important in breast carcinogenesis. By studying both preinvasive and invasive breast tumours, it should also be possible to gain further information about the relationship between lesions of a different stage and to determine whether DCIS is indeed a precursor of invasive ductal carcinoma. Levels of LOH were low in the invasive-only set of tumours. Surprisingly, considerably higher levels of loss were observed in the tumours with an in situ component. Also, much heterogeneity was observed between different DCIS ducts or invasive tumour and DCIS from the same case.

**Keywords:** ductal carcinoma in situ; microdissection; loss of heterozygosity; heterogeneity

Breast cancer remains one of the most common cancers in the Western world and overall it is estimated that approximately 1 in 10 women will develop this disease at some point in her life (Stratton and Wooster, 1996). Therefore, identification of environmental, biochemical and genetic factors that may be important in the aetiology and progression of this disease is essential in order to improve prevention, diagnosis and therapy. Conventionally, invasive cancer is regarded as developing through a series of events morphologically recognized as hyperplasia, atypical hyperplasia and in situ carcinoma, although for breast cancer this pathway of intermediate stages may be discontinuous and is certainly not universal. Although a variety of precursors of malignancy may be present in tissue surrounding invasive carcinoma, it is not uncommon for a cancer to be found in isolation, with no evidence of a non-infiltrating component in the area and no hint as to the nature of the progenitors of the disease. A close relationship between the preinvasive lesion ductal carcinoma in situ (DCIS) and invasive breast carcinoma has been postulated, as DCIS is frequently present in tissues adjacent to breast cancer (Ottesen et al, 1992; Schwartz et al, 1992). Furthermore, invasive breast cancers in women with DCIS generally occur in the same region of the same breast in which DCIS was originally identified (Lagios, 1993). By studying specific genetic alterations present in both preinvasive and invasive breast cancer, it was hoped to learn more about the relationship between these different stages.

In primary human breast tumours, loss of heterozygosity (LOH) represents the most frequent type of genetic alteration (Callahan et al, 1993). The assumption that LOH unmasks recessive alleles involved in tumorigenesis enables LOH analysis to be carried out on tumour DNA vs normal DNA, to determine the possible location of tumour-suppressor genes.

*Received 8 July 1997*
*Revised 6 October 1997*
*Accepted 20 October 1997*

*Correspondence to:* JM Varley

The chromosome region 9p21–p22 has attracted much research interest over recent years because of the identification, in 1994, of a new gene *p16* (also known as *MTS1*, for multiple tumour-suppressor 1, *INK4a* and *CDKN2*, for inhibitor of cyclin-dependent kinase). The discovery that the gene is homozygously deleted in a large number of human tumour cell lines (Kamb et al, 1994; Nobori et al, 1994) suggested that *p16* was behaving as a tumour-suppressor gene. The p16 protein had already been described as an inhibitor of cyclin-dependent kinases, which are themselves key regulators of the cell division cycle (Serrano et al, 1993). Loss of such an inhibitor would probably result in unregulated proliferation, thus reinforcing the support for *p16* as a tumour suppressor. More recently, the availability of the *p16–/–* mouse (Serrano et al, 1996) and the observation that some familial melanoma families harbour germline point mutations or deletions within the *p16* gene (Hussussian et al, 1994; Gruis et al, 1995) have provided further evidence that *p16* is a tumour-suppressor gene. A second gene in this region, *p15*, also behaves as a tumour suppressor (Sonada et al, 1995).

Regions of chromosome 9p have been found to be frequently rearranged or deleted in a range of primary tumours and often show LOH of genetic markers. There is evidence for a bladder cancer suppressor locus on chromosome 9p21 (Ruppert et al, 1993; Devlin et al, 1994; Orlow et al, 1994) with highest levels of loss at the *IFNA* gene cluster. Similar studies on other human tumours show significant LOH of the same region encompassing *IFNA*, including lung cancer (Mead et al, 1994; Merlo et al, 1994), glioma (Ichimura et al, 1994), renal cell carcinoma (Cairns et al, 1995) and oesophageal cancer (Tarmin et al, 1994). Some of these studies also describe high levels of loss both proximal and distal to *IFNA*, which are probably not targeting *p16*.

This study describes the assessment of LOH of microsatellite markers spanning chromosome 9p, to determine the location of putative tumour-suppressor genes involved in breast cancer.

**Table 1** Histology of tumours

| Case number | Histological classification | Nuclear grade |
|---|---|---|
| **DCIS** | | |
| 2652 | Comedo, solid | Intermediate |
| 2969 | Comedo, cancerization of lobules | Intermediate |
| 4419 | Comedo, cancerization of lobules, microinvasion | High |
| 6050 | Solid, micropapillary. Duct ectasia, tiny focus of invasion | High |
| 2238 | Comedo, cribriform. Lobular cancerization. Small focus of invasion | High |
| 2239 | Comedo, solid. Lobular cancerization | High |
| 2242 | Comedo, cribriform. Florid fibrocystic disease | High |
| 3812 | Comedo | Intermediate/low |
| 601 | Comedo. Occasional microinvasion | High |
| 6092 | Comedo. Small foci of invasion | High |
| 1960 | Solid. Fibrocystic change. Early microinvasion | High |
| 2659 | Comedo. Some stromal invasion | High |
| 4842 | Solid. Cancerization of lobules | Low |
| **DCIS + invasive** | | **Grade of DCIS/inv** |
| 3144 | Micropapillary. Invasive carcinoma. Fibrocystic disease | Intermediate/grade II |
| 6457 | Comedo, micropapillary. Invasive | High |
| 452 | Micropapillary. Infiltrating ductal carcinoma | High/grade II |
| 3041 | Cribriform, solid. Tubular carcinoma | Intermediate/grade I |
| 4410 | Comedo. Infiltrating ductal carcinoma | Intermediate/grade III |
| 6256 | Cribriform. Infiltrating ductal carcinoma | Intermediate/grade II |
| 6384 | Comedo. Infiltrating ductal carcinoma | High/grade II |
| 613 | Cribriform. Invasive carcinoma | Low |
| 2753 | Cribriform, solid. Infiltrating ductal carcinoma | Low–intermediate/II |
| 2931 | Micropapillary. Infiltrating ductal carcinoma | High/grade III |

Invasive cancers graded according to WHO guidelines. DCIS characterized using criteria based on nuclear grade.

### MATERIALS AND METHODS

### Tumour samples

Formalin-fixed, paraffin-embedded tumour tissue from 23 cases containing DCIS was obtained from Christie Hospital, Manchester, UK. Details of the histopathology and grade of these cases are given in Table 1. High molecular weight DNA had previously been prepared from frozen tumour material from a further 20 invasive breast cancer cases, for which there was also available the corresponding constitutional DNA, prepared from blood.

### Microdissection and DNA extraction

Areas of normal tissue, ducts containing DCIS and, where present, invasive carcinoma were microdissected from a single dewaxed 20-μm paraffin section, with reference to an adjacent haematoxylin- and eosin-stained section. Care was taken not to contaminate tumour cells with normal cells. DNA was extracted by standard methods (Sambrook et al, 1989). When this supply of DNA had been used, the same ducts and regions of tissue that had originally been taken were subsequently microdissected from the equivalent area on serial sections, to enable experiments to be repeated.

### PCR amplification

Primary PCRs were carried out in a volume of 20 μl, containing 340 μM each dNTP (Promega), 6 ng $\mu l^{-1}$ each of forward and reverse primer, 0.5–2 units Thermoprime plus DNA polymerase (Advanced Biotechnologies) and either 2–5 μl of DNA extracted from the microdissected tissue or 1 μl of blood–tumour DNA from the invasive breast cancer cases. Reactions were subjected to cycling conditions specific for each primer pair, with 25–50 cycles of denaturation at 94°C (typically 37 cycles for invasive tumour DNA and 43 cycles for DNA from microdissected material), annealing at temperatures of 55–62°C and extension at 72°C, each for 1 min. All polymerase chain reaction (PCR) programmes incorporated a denaturation step of 94°C for 4 min before cycling, with a final extension step of 72°C for 10 min after cycling.

An aliquot of 1 μl of product from the primary PCR was seeded into a 20-μl secondary PCR containing only one of the original primers, which had been end-labelled with [$\gamma^{32}$P]ATP using T4 polynucleotide kinase (200 ng of primer DNA and 1 μCi of ATP per reaction). The second round PCR programme consisted of just three cycles of 1 min at 94°C, 1 min at 55–62°C and 1 min at 72°C, followed by 2 min at 94°C, 5 min at 55–62°C and finally 10 min at 72°C.

### Oligonucleotide primers

Oligonucleotide primers described in Kwiatkowski and Diaz (1992), Weissenbach et al (1992), Wilkie et al (1992) and Gyapay et al (1994), were used to amplify microsatellite markers of the $(CA)_n$ repeat type. Relative positions of these markers are shown in Figure 1.

© Cancer Research Campaign 1998

**Figure 1** Human chromosome 9p, showing microsatellite markers. Consensus intermarker distances in cM, sex-averaged, taken from Kwiatkowski et al (1993) and Dib et al (1996). Markers originally identified by AFM code have been given their current D9S code (brackets)

### Detection of LOH

Radioactive PCR products were run on a denaturing 6% polyacrylamide gel at 75 W for 2–4 h. Dried gels were exposed to medical X-ray film that was developed by being placed in Kodak D19 developer and Ilford Hypam fixer followed by thorough washing with water. For each case the autoradiogram was studied and DNA from both normal and tumour material was assessed for differences in the intensity of the alleles between the two. Complete loss or reduction in intensity of over 40% (Hoggard et al, 1995) of one allele in the tumour relative to normal was considered to be LOH. For those cases that appeared to show loss of a particular marker, primary and secondary PCRs were repeated to confirm the result. To ensure that results were not caused by PCR artefacts, this was done using both the original DNA extract (when available) and DNA prepared from material freshly microdissected from the same duct on an adjacent serial section. A representative number of cases showing retention were also repeated, to confirm reproducibility of this technique.

### RESULTS

The results of the LOH study for each of the three separate tumour sets, invasive-only tumour, DCIS-only and invasive tumour with a DCIS component, are represented in Table 2. In addition, the percentage of informative ducts showing LOH was recorded for each marker (% loss).

### Invasive cases

As can be seen in Table 2a, eight of the twenty infiltrating carcinomas show loss of at least one marker on chromosome 9p. Of these cases, five show loss of more than one marker (129, 219, 330, 343 and 353), with regions of retention in between. This suggests the presence of interstitial deletions, rather than the complete loss of the short arm.

Only two tumours (330 and 343) show loss of one or both markers that flank *p16* (*IFNA* and D9S171). Case 330 demonstrates allelic imbalance at a number of markers across chromosome 9p, including a reduction in intensity of the lower allele at D9S285 (Figure 2A) and D9S1846/AFMb325zh9 (Figure 2B) and complete loss of the upper allele at markers *IFNA* and D9S171 (Figure 2C and D). The adjacent marker D9S1679/AFM337tb5 shows a reduction in intensity of the upper allele and there is retention of the more proximal marker D9S104 (Figure 2E and F). The fact that there is clear loss of the markers flanking *p16* indicates that the DNA template is relatively free from contaminating normal cells and that all cells may have undergone a targeted loss of *p16*. However, the presence of allelic imbalance rather than clear loss at other markers might suggest that only a proportion of cells carry a deletion of these particular markers. This could be explained if loss of the *p16* region was a relatively early event in the tumour's development, followed by the divergence of subclones, some of which undergo loss of other markers on chromosome 9p at a later stage.

Levels of loss at most other markers are also relatively low and may be explained by a background level of random genetic loss, which is generally considered to be approximately 15%. However, markers D9S1679 and D9S165 are lost in approximately 20% of informative cases and marker D9S285 is lost in 33% of cases, which may indicate the presence of putative tumour-suppressor genes in these regions.

### DCIS cases

In contrast to the results obtained for the invasive breast tumours, levels of LOH in the DCIS cases were significantly higher (Table 2b). Twelve of the thirteen cases studied showed loss of at least one marker on chromosome 9p. Each of the four subtypes of DCIS studied: comedo, cribriform, micropapillary and solid, were affected by loss of at least one marker, although the cases generally showing most extensive regions of loss, affecting multiple markers, were of the comedo or cribriform subtype. Case 2659 demonstrated loss of all informative markers that were tested, indicating loss of the complete short arm. A number of cases appeared to have undergone interstitial deletions, with retention of markers adjacent to regions of loss.

As the microdissection technique enabled precise removal of distinct ducts from the same tumour, it was possible to study the genetic alterations present in several separate ducts from the same case. It was thought that the comparison of such results would provide information on the clonality of these preinvasive lesions, for example the finding of the same genetic alteration in different ducts would support the idea that both ducts had developed from a common progenitor. However, this proved not to be the case and, of the ducts tested, a distinct pattern of allelic loss was observed in each.

Examples were found in which different ducts demonstrated imbalance of opposite alleles, suggesting that the ducts had undergone quite separate genetic alterations affecting different chromosomes. A number of comedo ducts from case 2238 were studied (Figure 3) and, taking the *IFNA* marker as an example, these ducts variously demonstrated retention of both alleles ($C_5$), loss of the lower allele ($C_2$) or loss of the upper allele ($C_1$ and $C_4$).

**Table 2** Results of loss of heterozygosity (LOH) studies on chromosome 9p in (a) invasive breast cancers, (b) DCIS and (c) cases with both DCIS and invasive cancer

(a) Invasive breast cancer cases

| Case / Locus | 76 | 127 | 129 | 139 | 161 | 163 | 166 | 219 | 228 | 330 | 343 | 344 | 353 | 364 | 369 | 370 | 374 | 389 | 395 | 413 | % Loss |
|---|---|---|---|---|---|---|---|---|---|---|---|---|---|---|---|---|---|---|---|---|---|
| D9S285 | – | ○ | | – | ○ | – | | – | | ● | ● | ○ | ● | – | – | – | ○ | ○ | ○ | | 33.3 |
| D9S156 | ○ | ○ | ○ | ○ | ○ | | | ○ | ○ | ○ | ○ | ○ | ○ | ○ | ● | | ○ | ○ | ● | ● | 17.6 |
| AFMa121yf1 (D9S1782) | ○ | – | | ○ | ○ | ○ | ○ | – | ○ | – | – | ○ | ○ | ○ | – | ○ | – | ○ | ○ | ○ | 0 |
| D9S162 | ○ | ○ | ● | ○ | ○ | ○ | ○ | ○ | – | – | ● | ○ | ○ | ○ | ○ | ○ | ○ | ○ | ○ | ○ | 11.1 |
| AFMb325zh9 (D9S1846) | ○ | ○ | ○ | ○ | – | ○ | – | ○ | – | ● | – | ○ | ● | ○ | ○ | ○ | ○ | ○ | ○ | ○ | 12.5 |
| *IFNA* | ○ | ○ | ○ | ○ | ○ | ○ | ○ | ○ | ○ | ● | ● | – | ○ | – | ○ | ○ | ○ | ○ | ○ | – | 11.8 |
| D9S171 | ○ | ○ | – | – | ○ | ○ | ○ | ○ | – | ● | – | ○ | ○ | ○ | – | ○ | ○ | – | – | ○ | 7.7 |
| AFM337tb5 (D9S1679) | ○ | ○ | ○ | ○ | – | ○ | ○ | ● | – | ● | ○ | – | ● | ○ | ○ | ○ | – | – | ○ | ○ | 20 |
| AFMa050yh1 (D9S1875) | – | ○ | ○ | – | ○ | ○ | ○ | ○ | – | ○ | ○ | ○ | ○ | ○ | – | – | ○ | ○ | – | ○ | 0 |
| D9S104 | ○ | ○ | – | ○ | – | ○ | ○ | ○ | ○ | ○ | ○ | – | – | – | – | ○ | ○ | – | ○ | – | 0 |
| D9S165 | ○ | ○ | ○ | – | ○ | ○ | ○ | ○ | – | ● | ● | ○ | ● | – | ○ | – | ○ | ○ | ○ | ○ | 18.75 |
| AFM155xh4 (D9S1788) | – | | ● | | ○ | ○ | | ○ | | ○ | – | ○ | ○ | ○ | ○ | ○ | ○ | ○ | ○ | ○ | 7.1 |
| D9S166 | ○ | – | ○ | – | ○ | ○ | ○ | ● | ○ | ○ | – | ○ | ○ | ○ | ○ | ○ | – | ○ | – | | 7.1 |

(b) DCIS cases

| Case / Locus | 2652 | | 2969 | | 4419 | | 6050 | | 2238 | | | | | | 2239 | | 2242 | | 3812 | 601 | | 6092 | 1960 | | 2659 | 4842 | | % Loss |
|---|---|---|---|---|---|---|---|---|---|---|---|---|---|---|---|---|---|---|---|---|---|---|---|---|---|---|---|---|
| | C | S | C1 | C2 | C1 | C2 | M | S | C1 | C2 | C3 | C4 | C5 | Cr | C | S | Cr | C | C | C1 | C2 | C | S1 | S2 | C | S1 | S2 | |
| D9S285 | ● | | ○ | | | ○ | | | | | | | | | ○ | ○ | ● | | ● | ○ | ● | ○ | | | ● | | | 45.5 |
| D9S156 | ○ | | | | ○ | ○ | ○ | ○ | | | | | | | | | ● | | ● | – | – | | | | | | | 28.6 |
| AFMa121yf1 (D9S1782) | | | | | | | | | | | | | | | – | – | | | | | | – | – | – | | | | 0 |
| D9S162 | ● | | ○ | ● | ● | | ● | ○ | ○ | ● | ○ | | | | ○ | ○ | ○ | | – | ○ | ● | – | | | – | | ○ | 40 |
| AFMb325zh9 (D9S1846) | – | – | ○ | | – | – | ● | | | | | | | | ● | | | ● | | ○ | ● | – | ○ | ○ | – | | | 50 |
| *IFNA* | ● | ○ | – | – | – | – | – | – | ● | ●′ | | ●′ | ○ | | ○ | | ● | ○ | ○ | ● | | – | | | ● | | | 50 |
| D9S171 | ● | ○ | | ○ | | | ○ | | – | – | – | – | – | – | – | – | – | – | | | | ○ | ● | | | | | 33.3 |
| AFM337tb5 (D9S1679) | | | – | – | | | ○ | | | | | | | | ○ | ● | | ● | ● | – | – | – | ○ | ● | | ○ | | 50 |
| AFMa050yh1 (D9S1875) | | | | | | | | | | | | | | | | ○ | | ● | ● | | | ○ | | ○ | – | | | 40 |
| D9S104 | ● | ○ | ● | | – | – | ○ | ○ | ○ | | ● | | | ○ | ○ | ○ | – | – | | – | – | ○ | ○ | ● | ● | | | 35.7 |
| D9S165 | | | | ● | | | | | | ● | | | | | | ○ | | ○ | | | | ○ | | ○ | ● | | | 42.9 |
| AFM155xh4 (D9S1788) | ● | | ● | ○ | | ● | ○ | | ○ | | ● | | | ○ | ● | ○ | ○ | ○ | | – | – | ○ | ○ | ○ | ● | ● | ○ | 38.9 |

© Cancer Research Campaign 1998

**Table 2** (Cont)

(c) DCIS/invasive cases

| Case<br>Locus | 3144<br>M | <br>I | 6457<br>C1 | <br>C2 | <br>C3 | <br>C4 | <br>M | <br>I | 452<br>M | <br>I | 3041<br>Cr | <br>S | <br>I | 4410<br>C | <br>I | 6256<br>Cr | <br>I | 6384<br>C | <br>I | 613<br>Cr 1 | <br>Cr 2 | <br>I | 2753<br>C | <br>S | <br>I | 2931<br>M | <br>I | % Loss |
|---|---|---|---|---|---|---|---|---|---|---|---|---|---|---|---|---|---|---|---|---|---|---|---|---|---|---|---|---|
| D9S285 | | | ○ | | | ○ | ● | | | | ○ | | ○ | ● | ● | | | | | | ○ | | | | | | | 37.5 |
| D9S156 | | | | | | ○ | ● | | | | | | | – | – | | | | | | | | | | | | | 50 |
| AFMa121yf1 (D9S1782) | | | – | – | – | – | – | – | | | | | | | | – | – | | | – | – | – | | | | | | 0 |
| D9S162 | – | – | ○ | | | ○ | ● | ○ | – | – | | | ● | ● | ● | – | – | ● | ● | ● | ○ | | ● | ○ | | ○ | | 57.1 |
| AFMb325zh9 (D9S1846) | – | – | ○ | | | ○ | ○ | ○ | | | | | | ● | ● | | ○ | | | ● | | | – | – | – | | | 37.5 |
| IFNA | | ○ | ○ | ● | ○ | ○ | ● | ○ | | | | ● | ○ | | | ○ | ● | – | – | ● | | | | | | | | 41.7 |
| D9S171 | | | | | | ○ | ○ | ○ | | | | | ○ | – | – | | ○ | | | | | | | | | | | 0 |
| AFM337tb5 (D9S1679) | ○ | | – | – | – | – | – | – | | | | | | ● | ● | – | – | | | | | | – | – | – | | | 66.7 |
| AFMa050yh1 (D9S1875) | | ● | – | – | – | – | – | – | | | | | | | | | | | | ● | | | | | | | | 100 |
| D9S104 | | ○ | ○ | ●′ | ●′ | ○ | ○ | ○ | | | | ● | ● | ○ | ○ | ○ | ○ | – | – | | | | | ○ | | | | 28.6 |
| D9S165 | | | ○ | | | | | ● | | | | | | | | | ● | | | – | – | – | | | | | | 66.7 |
| AFM155xh4 (D9S1788) | | ● | ○ | | | ○ | ○ | ○ | ○ | ○ | ○ | | ○ | ● | ● | ● | ● | | | ○ | | | ● | ● | ● | ○ | ○ | 42.1 |

(○), Informative and both alleles retained; (●), informative and showing allelic imbalance; (–) not informative, i.e. homozygous; no symbol, lack of material or consistent failure to amplify; (′) different alleles lost in different ducts. DCIS subtypes: M micropapillary, C comedo, Cr cribriform, S solid, I invasive.

**Figure 2** Case 330 showing clear loss of the upper allele at the *IFNA* and D9S171 markers in the invasive DNA (I) compared with the normal DNA (N). Allelic imbalance can be seen in the flanking markers (**A**), (**B**) and (**E**), with retention of both alleles at marker (**F**) D9S104

**Figure 3** Several different comedo ducts taken from case 2238 show evidence of having undergone distinct genetic alterations. Duct $C_5$ shows retention of both alleles of the *IFNA* marker. Duct $C_2$ shows loss of the lower allele in contrast to duct $C_4$, which shows loss of the upper allele. Duct $C_1$ shows allelic imbalance with a reduction in intensity of the upper allele. *Cases showing LOH

Six of the thirteen DCIS cases studied demonstrated LOH of at least one marker flanking *p16* (*IFNA* or D9S171) that could potentially have arisen as a result of targeted loss of *p16* accompanied by loss of additional markers. The *IFNA* marker was lost in 50% of ducts examined. High levels of loss comparable with this were also found at markers both proximal and distal to *p16*. As had been observed in the invasive cases, markers D9S165 and D9S285 were among those affected with the highest frequency, being lost in > 40% of cases. However, D9S1846 and D9S1679, like *IFNA*, were lost in 50% of cases, although the number of ducts assessed was only relatively small in each case. Again, these high levels of loss may indicate the location of putative tumour-suppressor genes.

### DCIS cases with an invasive component

A number of markers spanning chromosome 9p were successfully amplified from both comedo DCIS DNA (C) and infiltrating ductal carcinoma DNA (I) from case 4410. As the results in Table 2c and Figure 4 show, concordance between these two components was found in every case. These results suggest that, in this case at least, the DCIS and the invasive tumours are intimately related, the most likely explanation being that the DCIS was the precursor lesion. However, a more complex pattern of genetic alterations is observed in other cases, with the preinvasive and invasive components not showing the same changes. For example case 6256 demonstrates retention of both alleles of the *IFNA* marker in the cribriform DCIS but loss of the upper allele in the invasive component, perhaps indicating that loss of this particular region is important in the progression from in situ to invasive carcinoma (Figure 5). A slightly more complex situation is shown in Figure 6. As had been observed in the subgroup of tumours containing DCIS only, when numerous ducts were microdissected from cases containing both DCIS and carcinoma, retention of both alleles was found in some ducts, with LOH in others. Also, as noted previously, losses in different ducts may affect different alleles, reinforcing the idea that DCIS shows considerable genetic heterogeneity.

© Cancer Research Campaign 1998

**Figure 4** Case 4410 showing concordance between genetic alterations observed in the comedo DCIS (C) and invasive components (I). LOH is observed in both preinvasive and invasive tumour at every marker tested, including (**A**), (**B**), (**C**) and (**E**), except marker (**D**) D9S104, which shows retention in both C and I

### DISCUSSION

Despite the uncertainty regarding the relevance of losses flanking *p16*, the levels of loss at other markers on chromosome 9p, in the DCIS and DCIS/invasive cases in particular, suggested the possible location of other tumour-suppressor genes both proximal and distal to *p16*. D9S285 was lost at consistently high frequency (> 30%) across the three tumour sets, as were markers D9S1679 and D9S165. Also, although levels of loss in the invasive panel of tumours were low, the markers D9S1788 and D9S162 were lost in approximately 40% or more of cases in the remaining two groups of tumours. Other studies looking at LOH of microsatellite markers spanning chromosome 9p in various tumours have found similar results, with many losses centred around the *IFNA* marker, but also extending proximally and distally (Olopade et al, 1993; Coleman et al, 1994; Neville et al, 1995).

One purpose of the study had been to attempt to gain more information about the natural history of breast cancer and the molecular changes accompanying progression. This study and

**Figure 5** Possible genetic alterations accompanying progression. Case 6256 showing retention of both alleles of marker *IFNA* in the cribriform DCIS (Cr) and loss of the upper allele (*) in the invasive component (I). This may indicate that deletion of this region of chromosome 9p is important in the progression to invasive ductal carcinoma others (James et al, 1997) have confirmed that genetic changes found in DCIS lesions are similar to those found in invasive breast carcinoma. Furthermore, a recent paper has also described some changes in morphologically normal tissue adjacent to breast carcinomas (Deng et al, 1996).

One significant conclusion to be drawn from this work is that, even at the preinvasive stage of DCIS, breast cancer is a heterogeneous disease in which the pathway of changes accompanying progression is far from clear. Although the results indicated that some DCIS lesions are likely to be the precursors of invasive cancer, because both components showed identical allele loss for particular markers, this is not true for all DCIS, some of which have quite distinct genetic alterations to those of the invasive component. Reports in the literature have described both situations. A study by Zhuang et al (1995) assessed allelic imbalance of markers on chromosome 11q13 in microdissected in situ and invasive human breast cancer. This provided evidence for a tumour-suppressor gene in this region that may be important in breast cancer development, and also indicated that invasive breast cancer arises from in situ lesions. However, similar studies looking at allelic imbalance have described intratumour heterogeneity with the probable existence of subpopulations of tumour cells (Chen et al, 1992; Bonsing et al, 1993). An extension of this has been noted in studies similar to this one, in which several ducts from the same case were treated independently. In agreement with the results described in this study, heterogeneity with respect to allele loss was found in different neoplastic foci of in situ cancer and in some cases allelic losses present in in situ regions were not conserved in the progression to invasive tumour (Munn et al, 1995; 1996; Fujii et al, 1996).

It remains surprising that the degree of loss observed in the invasive-only set of tumours was so much less than that found in the other two sets. It might be expected that because the DCIS lesions show such a high level of loss of markers on chromosome 9 that they may show a correspondingly high frequency of loss of other markers across the genome. However, this has been shown not to be the case (Aldaz et al, 1995; Radford et al, 1995), with allelotype studies on DCIS showing levels of loss less than those found in invasive breast carcinomas. Although direct comparisons between the three sets of tumours cannot be made and numbers are small, the results of this study may suggest that this particular set of invasive lesions is fundamentally different to those lesions that have an intraductal component. A similar result has previously

© Cancer Research Campaign 1998 

**Figure 6** Both loss and retention of the same marker in different ducts from the same case. (**A**) The comedo duct (C) from case 6457 shows retention of the upper allele of marker D9S162 in contrast to the micropapillary duct (M), which shows clear loss of this allele. Similarly, in case 2753 clear LOH is seen in the cribriform duct (Cr), in contrast to the retention of both alleles seen in the solid (S) duct. (**B**) This phenomenon is not confined to ducts of different subtype, as different comedo ducts from case 6457 show different results when the genotype of the *IFNA* marker is assessed. (**C**) A further level of heterogeneity exists between individual ducts from the same case. Case 6457 demonstrates retention of both alleles at marker D9S104 in duct $C_4$. However, ducts $C_3$ and $C_2$ show loss of opposite alleles, indicating that they have undergone distinct genetic alterations affecting different chromosomes been obtained from immunohistochemical studies of the protein encoded by the c-*erb*B-2 oncogene (Barnes et al, 1992). Despite associations between amplification/overexpression of this oncogene and poor prognosis in invasive breast tumours, these studies showed much higher levels of overexpression in preinvasive comedo DCIS than infiltrating breast carcinomas. This could be explained if there existed a subgroup of invasive tumours that, despite a lack of c-*erb*B-2 positivity, were still associated with poor prognosis. DCIS cases showing staining for this oncoprotein were also associated with a greater invasive potential, but it was suggested that for those invasive cancers that did not show staining, their high rate of proliferation and associated malignant potential was possibly correlated with particular characteristics of nuclear grade. Such cases were expected to have only a transient progression through a preinvasive DCIS stage, which would probably not be observed as negative staining in DCIS. Hence, relatively fewer DCIS cases than invasive tumours would be negative for c-*erb*B-2 staining. Similarly, in this study, alterations to chromosome 9p may be important in the progression to neoplasia of a subgroup of tumours, although, in the absence of such alterations, as in the invasive carcinomas studied, other features must be considered.

### ACKNOWLEDGEMENTS

This work was supported by the Cancer Research Campaign and KLM was in receipt of a sponsorship from Zeneca Pharmaceuticals. We would like to thank Dr Lia Menasce for confirming the nuclear grade of the DCIS cases.

### REFERENCES

Aldaz CM, Chen T, Sahin A, Cunningham J and Bondy M (1995) Comparative allelotype of *in situ* and invasive human breast cancer: high frequency of microsatellite instability in lobular breast carcinomas. *Cancer Res* **55**: 3976–3981

Barnes DM, Bartkova J, Camplejohn RS, Gullick WJ, Smith PS and Millis RR (1992) Overexpression of the c-*erb*B-2 oncoprotein: why does this occur more frequently in ductal carcinoma *in situ* than in invasive mammary carcinoma and is this of prognostic significance? *Eur J Cancer* **28**: 644–648

Bonsing BA, Devilee P, Cleton-Jansen A-M, Kuipers-Dijkshoorn N, Fleuren GJ and Cornelisse CJ (1993) Evidence for limited molecular genetic heterogeneity as defined by allelotyping and clonal analysis in nine metastatic breast carcinomas. *Cancer Res* **53**: 3804–3811

Cairns P, Tokino K, Eby Y and Sidransky D (1995). Localisation of tumour suppressor loci on chromosome 9 in primary human renal cell carcinoma. *Cancer Res* **55**: 224–227

Callahan R, Cropp C, Sheng ZM, Merlo G, Steeg P, Liscia D and Lidereau R (1993) Definition of regions of the human genome affected by loss of heterozygosity in primary human breast tumors. *J Cell Biochem* **17G**: 167–172

Chen LC, Kurisu W, Ljung BM, Goldman ES, Moore D and Smith HS (1992) Heterogeneity for allelic loss in human breast cancer. *J Natl Cancer Inst* **84**: 506–510

Coleman A, Fountain JW, Nobori T, Olopade OI, Robertson G, Housman DE and Lugo TG (1994) Distinct deletions of chromosome 9p associated with melanoma versus glioma, lung cancer, and leukemia. *Cancer Res* **54**: 344–348

Deng G, Lu Y, Zlotnikov G, Thor AD and Smith HS (1996) Loss of heterozygosity in normal tissue adjacent to breast carcinomas. *Science* **274**: 2057–2059

Devlin J, Keen AJ and Knowles MA (1994) Homozygous deletion mapping at 9p21 in bladder carcinomas defines a critical region within 2 cM of *IFNA: Oncogene* **9**: 2757–2760

Dib C, Faure S, Fizames C, Samson D, Drouot N, Vignal A, Millasseau P, Marc S, Hazan J, Seboun E, Lathrop M, Gyapay G, Morissette J and Weissenbach J (1996) A comprehensive genetic map of the human genome based on 5,264 microsatellites. *Nature* **380**: 152–154

Fujii H, Marsh C, Cairns P, Sidransky D and Gabrielson E (1996) Genetic divergence in the clonal evolution of breast cancer. *Cancer Res* **56**: 1493–1497

Gruis NA, Van Der Velden PA, Sandkuijl LA, Prins DE, Weaver-Feldhaus J, Kamb A, Bergman W and Frants RR (1995) Homozygotes for *CDKN2 (p16)* germline mutation in Dutch familial melanoma kindreds. *Nature Genet* **10**: 351–353

Gyapay G, Morissette J, Vignal A, Dib C, Fizames C, Millasseau C, Marc S, Bernadi G, Lathrop M and Weissenbach J (1994) The 1993–1994 Genethon human genetic linkage map. *Nature Genet* **7**: 246–339

Hoggard N, Brintnell B, Howell A, Weissenbach J and Varley J (1995) Allelic imbalance on chromosome 1 in human breast cancer. II. Microsatellite repeat analysis. *Genes Chromosom Cancer* **12**: 24–31

© Cancer Research Campaign 1998

Hussussian CJ, Struewing JP, Goldstein AM, Higgins PA, Ally DS, Sheahan MD, Clark WH, Tucker MA and Dracopoli NC (1994) Germline *p16* mutations in familial melanoma. *Nature Genet* **8**: 15–21

Ichimura K, Schmidt EE, Yamaguchi N, James CD and Collins VP (1994) A common region of homozygous deletion in malignant human gliomas lies between the *IFNa/w* gene cluster and the *D9S171* locus. *Cancer Res* **54**: 3127–3130

James LA, Mitchell ELD, Menasce L and Varley JM (1997) Comparative genomic hybridisation of ductal carcinoma in situ of the breast: identification of regions of DNA amplification and deletion in common with invasive breast carcinoma. *Oncogene* (in press)

Kamb A, Gruis NA, Weaver-Feldhaus J, Liu Q, Harshman K, Tavtigian SV, Stockert E, Day RSI, Johnson BE and Skolnick MH (1994) A cell cycle regulator potentially involved in genesis of many tumour types. *Science* **264**: 436–440

Kwiatkowski DJ and Diaz MO (1992) Dinucleotide repeat polymorphism at the IFNA locus (9p22). *Hum Mol Genet* **1**: 658

Lagios MD (1993) Heterogeneity of ductal carcinoma *in situ* of the breast. *J Cell Biochem* **17G**: 49–52

Mead LJ, Gillespie MT, Irving LB and Campbell LJ (1994) Homozygous and hemizygous deletions of 9p centromeric to the interferon genes in lung cancer. *Cancer Res* **54**: 2307–2309

Merlo A, Gabrielson E, Mabry M, Vollmer R, Baylin SB and Sidransky D (1994) Homozygous deletion on chromosome 9p and loss of heterozygosity on 9q, 6p and 6q in primary human small cell lung cancer. *Cancer Res* **54**: 2322–2326

Munn KE, Walker RA and Varley JM (1995) Frequent alterations of chromosome 1 in ductal carcinoma *in situ* of the breast. *Oncogene* **10**: 1653–1657

Munn KE, Walker RA, Menasce L and Varley JM (1996) Allelic imbalance in the region of the *BRCA*1 gene in ductal carcinoma *in situ* of the breast. *Br J Cancer* **73**: 636–639

Neville EM, Stewart M, Myskow M, Donnelly RJ and Field JK (1995) Loss of heterozygosity at 9p23 defines a novel locus in non-small cell lung cancer. *Oncogene* **11**: 581–585

Nobori T, Miura K, Wu DJ, Lois A, Takabayashi K and Carson DA (1994) Deletions of the cyclin-dependent kinase-4 inhibitor gene in multiple human cancers. *Nature* **368**: 753–756

Olopade OI, Buchhagen DL, Malik K, Sherman J, Nobori T, Bader S, Nau MM, Gazdar AF, Minna JD and Diaz MO (1993) Homozygous loss of the interferon genes defines the critical region on 9p that is deleted in lung cancers. *Cancer Res* **53**: 2410–2415

Orlow I, Lianes P, Lacombe L, Dalbagni G, Reuter VE and Cordon-Cardo C (1994) Chromosome 9 allelic losses and microsatellite alterations in human bladder tumours. *Cancer Res* **54**: 2848–2851

Ottesen GL, Graversen HP, Blichert TM, Zedeler K and Andersen JA (1992) Ductal carcinoma in situ of the female breast: short-term results of a prospective nationwide study. *Am J Surg Pathol* **16**: 1183–1196

Radford DM, Fair KL, Phillips NJ, Ritter JH, Steinbrueck T, Holt MS and Donis-Keller H (1995) Allelotyping of ductal carcinoma in situ of the breast: deletion of loci on 8p, 13q, 16q, 17p and 17q. *Cancer Res* **55**: 3399–3405

Ruppert JM, Tokino K and Sidransky D (1993) Evidence for two bladder cancer suppressor loci on human chromosome 9. *Cancer Res* **53**: 5093–5095

Sambrook J, Fritsch EF and Maniatis T (1989) *Molecular Cloning – A Laboratory Manual*, 2nd edn. Cold Spring Harbor Laboratory Press.

Schwartz GF, Finkel GC, Garcia JC and Patchefsky AS (1992) Subclinical ductal carcinoma in situ of the breast. *Cancer* **70**: 2468–2474

Serrano M, Hannon GJ and Beach D (1993) A new regulatory motif in cell-cycle control causing specific inhibition of cyclin D/CDK4. *Nature* **366**: 704–707

Serrano M, Lee H-W, Chin L, Cordon-Cardo C, Beach D and Depinho RA (1996) Role of the *INK4a* locus in tumour suppression and cell mortality. *Cell* **85**: 27–37

Sonada Y, Yashimoto T and Sekiya T (1995) Homozygous deletion of the *MTS1/p16* and *MTS2/p15* genes and amplification of the CDK4 gene in glioma. *Oncogene* **11**: 2145–2149

Stratton MR and Wooster R (1996) Hereditary predisposition to breast cancer. *Curr Opin Genet Dev* **6**: 93–97

Tarmin L, Yin J, Zhou X, Suzuki H, Jiang H-Y, Rhyu M-G, Abraham JM, Krasna MJ, Cottrell J and Meltzer SJ (1994). Frequent loss of heterozygosity on chromosome 9 jn adenocarcinoma and squamous cell carcinoma of the oesophagus. *Cancer Res* **54**: 6094–6096

Weissenbach J, Gyapay G, Dib C, Vignal A, Morissette J, Millasseau P, Vaysseix G and Lathrop M (1992) A second-generation linkage map of the human genome. *Nature* **359**: 794–801

Wilkie PJ, Krizman DB and Weber JL (1992) Linkage map of human chromosome 9 microsatellite polymorphisms. *Genomics* **12**: 607–609

Zhuang Z, Merino MJ, Chuaqui R, Liotta LA and Emmert-Buck MR (1995) Identical allelic loss on chromosome 11q13 in microdissected in situ and invasive human breast cancer. *Cancer Res* **55**: 467–471

© Cancer Research Campaign 1998